# MiRNAs as New Tools in Lesion Vitality Evaluation: A Systematic Review and Their Forensic Applications

**DOI:** 10.3390/biomedicines9111731

**Published:** 2021-11-20

**Authors:** Alice Chiara Manetti, Aniello Maiese, Arianna Baronti, Eleonora Mezzetti, Paola Frati, Vittorio Fineschi, Emanuela Turillazzi

**Affiliations:** 1 Department of Surgical, Medical and Molecular Pathology and Critical Care Medicine, Section of Legal Medicine, University of Pisa, 56126 Pisa, Italy; a.manetti3@studenti.unipi.it (A.C.M.); aniello.maiese@unipi.it (A.M.); a.baronti@studenti.unipi.it (A.B.); e.mezzetti1@studenti.unipi.it (E.M.); emanuela.turillazzi@unipi.it (E.T.); 2 Department of Anatomical, Histological, Forensic and Orthopedic Sciences, Sapienza University of Rome, 00186 Rome, Italy; paola.frati@uniroma1.it

**Keywords:** miRNAs, lesion vitality, wound forensic, genetic

## Abstract

Wound vitality demonstration is one of the most challenging fields in forensic pathology. In recent years, researchers focused on the application of histological and immunohistochemical staining in this sphere of study. It is based on the detection of inflammation, red cell infiltration, and tissue alterations at the histological examination, all of which are supposedly present in antemortem rather than post-mortem wounds. Nevertheless, some doubts about the reliability of those markers have arisen. Furthermore, the lack of a standardized protocol and the operator dependency of this approach make the proper interpretation of its results difficult. Moreover, a differential miRNAs expression has been demonstrated in antemortem and post-mortem wounds. Herein, a systematic review concerning the current knowledge about the use of miRNAs in lesion vitality evaluation is carried out, to encourage researchers to deepen this peculiar study area. A compendium about the potential miRNAs that may be further investigated as vitality markers is also provided. The aim is to collect all available data about this topic to direct further studies on this field and highlight the future applications of miRNAs in forensic pathology. We found 20 articles and a total of 51 miRNAs that are involved in inflammation and wound healing. Further studies are certainly needed to deepen the role of miRNAs in inflammatory processes in lesioned skin and to evaluate their reliability in distinguishing between antemortem and post-mortem lesions.

## 1. Introduction

Wound vitality demonstration is one of the most challenging fields in forensic pathology. It has been classically based on the presence of inflammation, red cell infiltration, and tissue alterations at the histological examination [1,2,3,4]. However, the differentiation between vital reactions and post-mortem changes is not always clear [5,6,7]. In recent years, researchers focused on the detection of inflammatory cells, cytokines, apoptosis mediators, and other markers that are supposedly present in antemortem rather than post-mortem wounds, through immunohistochemical stains [6,7,8,9,10,11]. Nevertheless, some doubts about the reliability of these methods have arisen, and the lack of a standardized protocol and the operator dependency of this approach makes it difficult to correctly interpret the results [12].

MicroRNAs (miRNAs) are short single-strand non-coding ribonucleic acids that have a fundamental role in the post-transcriptional regulation of gene expression, mainly inhibiting the mRNA translation [13,14,15]. Due to their ubiquity, they can be used as a diagnostic tool in different clinical areas, as well as in forensic pathology [16]. A differential miRNA expression in antemortem and post-mortem wounds has been observed [17,18,19]. This evidence opens new and promising possibilities to use them as lesion vitality markers. It is unlikely that a method alone could provide the trustworthiness needed in judicial cases, but the combination of histological, immunohistochemical, and genetic analysis may become a new tool in vitality diagnosis.

## 2. Materials and Methods

The present systematic review was carried out according to the Preferred Reporting Items for Systematic Review (PRISMA) standards [20]. A systematic literature search and a critical review of the collected studies were conducted. An electronic search of PubMed, Science Direct Scopus, Google Scholar, and Excerpta Medica Database (EMBASE) from database inception to November 2020 was performed. The search terms were “miRNAs”, “wound vitality”, and “lesions vitality” in the title, abstract, and keywords. The bibliographies of all located papers were examined and cross-referenced to further identify relevant literature. A methodological appraisal of each study was conducted according to the PRISMA standards, including an evaluation of bias. The data collection process included study selection and data extraction. Two researchers (A.C.M., E.M.) independently examined the papers with titles or abstracts that appeared to be relevant and selected those that analyzed miRNAs involved in wound vitality demonstration and miRNAs involved in wound healing that may be further investigated as vitality markers in post-mortem samples. Researchers resolved their disagreement concerning works eligibility by consensus. Only papers in English were included in the research. Two investigators performed data extraction (A.B., E.M.), and two other investigators verified them (A.B., E.M.), which were again verified by two other investigators (A.M., E.T.). This study was exempt from institutional review board approval, as it did not involve human subjects.

## 3. Results

The search performed as described above identified 197 articles, which were screened to exclude duplicates. The resulting 163 reference lists were then screened based on their title and abstract, which left 87 articles for further consideration. Non-English papers were excluded. The following inclusion criteria were used: (1) original research articles, (2) reviews and mini-reviews, and (3) case reports/series. These publications were carefully evaluated, considering the main aims of the review. This evaluation left 20 scientific papers comprising original research articles, case reports, and case series.

**Figure 1:**
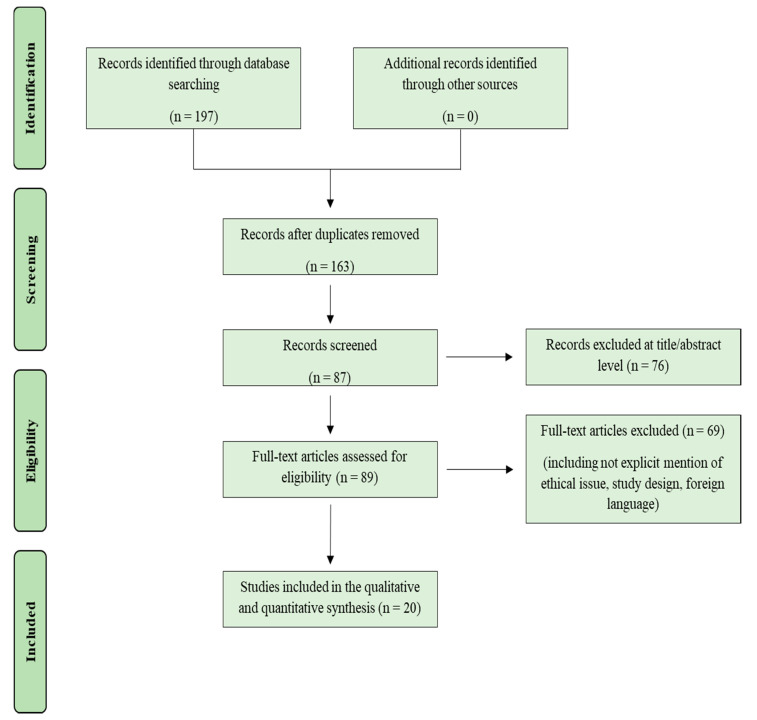
Search strategy following PRISMA standards, including an evaluation of bias.

Figure 1 illustrates our search strategy.

Table 1 shows the studies included in this review with a brief description.

As shown in Table 2, which summarizes the main characteristics of the articles included in this review, most of the founded studies are about miRNAs involved in wound healing and therefore they are based on in vitro and/or in vivo experiments [17,18,21,22,23,24,25,26,27,28,29,30,31,32,33,34,35,36,37]. 

Three studies used human skin lesion samples collected post-mortem (2) and antemortem (1) [19,29,37]. Animal studies were based on antemortem samples, mainly collected within a precise interval after wounding (days), while antemortem human skin samples were hypertrophic skin scars specimens collected during surgical procedures. In vitro and antemortem studies investigated the role of miRNAs in wound healing and, as previously mentioned, they were included in this review because they could suggest new miRNAs to study for wound vitality demonstration in post-mortem samples. Moreover, antemortem studies provide new knowledge about the expression of miRNAs in wound healing in different timeframes, and consequently, they may be used in further studies to determine the age of a lesion. Concerning the type of skin lesion, the articles investigated miRNAs expression in incisional [18] and excisional wound [21,22,23,26,27,30,31,33,34,35], ligature mark [19], burned skin [37], chronic ulcer [32], and hypertrophic scar [29]. There is heterogeneity among researchers concerning the type of adopted investigation. Real-time polymerase chain reaction (RT-PCR) was performed in 17 studies out of 20. In 11 studies, Western blot was used for protein analysis [21,22,24,25,26,27,28,30,32,33,36]. Histological examination of the wounded skin was performed in 13 out of 20 studies, but only in 8 studies, this evaluation was supplemented by immunohistochemical analysis and in 5 by hybridization in situ [17,18,19,21,22,23,24,25,27,28,29,32,33,34,35,36]. Immunofluorescence analysis was performed in two studies, while a morphometric analysis appeared in the work of Long and coll [31]. Flow cytometry analysis was used by Lin et al. to isolate fat cells [28]. Phase-contrast morphometry was performed in one study [36]. In all the studies included in this review, a statistical analysis on obtained results was performed, except for Viticchiè et al., Yang et al., and Yu et al. [33,35,36].

As a result of our research, we found that 51 miRNAs are implied in wound healing and wound vitality demonstration. To understand the molecular pathways in which these miRNAs are involved, we collected their target genes and/or proteins, when specified by the included papers, as shown in Table 3.

Some miRNAs have a differential expression over time after wounding. This “day-by-day” variation may help determine the age of a lesion. According to the studies performed by Lin et al. and Yu et al., miR-205 shows an increase during the first days in epithelial cells, while on the contrary, Etich et al. found that it is downregulated from day 1 to day 10 after wounding [23,28,36]. MiR-483-3p, studied by Bertero et al., starts to increase 3 days after the production of the wound and leads to its peak at 6–7 days [21]. MiR-21 seems to rise, and Yang et al. demonstrated its increase over three days [35]. In the presence of H_2_O_2_, both miR-19b and miR-124 levels decline [22,24]. Etich et al. proved the downregulation of miR-204 and miR-205 (respectively, from day 5 to 10, and from day 1 to day 7), and the contemporaneous overexpression of miR-31 [23]. Moreover, Wang et al. attested mir-31 increase, along with miR-21, and miR-203; miR-249 is the only one that decreases 7 days after injuring [34]. Another investigation about miR-16, miR-20a, miR-106a, miR-130a, and miR-203 in chronic ulcers revealed their growth in the epidermis [32]. In the same investigation, miR-21 levels were found overexpressed in all skin layers. Similarly, miR-152, miR-365, miR-125a-5p, miR-181d, miR-99, miR-100, miR-30c, and miR-125b-5p were downexpressed from day 1, returning to normal levels after five days [26]. A similar trend has been described for miR-149, miR-203a, miR-222, and miR-122 [29]. Lyu et al. proved the overexpression of 19 different miRNAs and the simultaneous decrease in five miRNAs in burned skin [17]. In another experiment conducted on burned skin, the authors established the upregulation of miR-711 and miR-183-3p 48 h after excision in human skin [37]. The same experiment, performed on mice burned skin by the same Authors, revealed that their rising occurred up to 120 h after wounding. MiR-203 has been investigated in five studies, which showed it is increased after wounding except in one case, where a decrease in the epidermis, dermis, and subcutaneous fat over four days was demonstrated [29,30,32,33,34]. Neri et al. studied the expression of five different miRNAs in ligature marks, showing the elevation of miR125a-5, miR125b-5, miR130a-3, miR214-3p, and miR92a-3p [19]. 

In summary, during the first day after wounding, it is possible to find the overexpression of at least 10 different miRNAs (miR-205, miR-152, miR-365, miR-125a-5p, miR-181d, MiR-99, miR-100, miR-30c, miR-125b-5p, and miR203) and the contemporaneous decreasing of miR-21. On day 2, miR-205, miR-21, miR-711, and miR-183-3p levels increase. The expression of miR-483-3p, miR-203, and miR-21 rises on day 3. From the fifth day, miR-31 and miR-203 levels rise, while miR-204 expression decreases. Eventually, from day 7, miR-249 starts to decrease.

## 4. Discussion

MiRNAs have attracted the attention of researchers in various fields [38,39,40,41]. They influence many signaling pathways involved in inflammation [42,43,44,45]. Due to this property, miRNAs could be used in vitality wound demonstration. In this review, we present many miRNAs that have a role in wound healing and, therefore, may be investigated as post-mortem vitality markers. 

Regarding the molecular pathways involved in the inflammation and healing processes highlighted in this work, miR-19-b regulates the transforming growth factor-β (TGF-β) signaling pathway by targeting C-C motif chemokine ligand 1 (CCL1) [46]. The TGF-β pathway, determinant in wound contraction, can also be modified by miR-21 and miR-149 [27,34]. Under inflammatory conditions, miR-149 also inhibits the interleukin (IL) 1α, IL-1β, and IL-6 genes, as well as the nuclear factor kappa-light-chain-enhancer of activated B cells (NF-κB) pathway [47]. NFκB pathway is activated by the tumor necrosis factor (TNFα) phosphorylating the inhibitor of nuclear factor kappa B (IκB) [48]. In keratinocytes, MiR-205 and miR-184 interact in regulating the SH2-containing phosphatidylinositol 3,4,5-trisphosphate 5-phosphatase (SHIP2) levels and phosphorous protein kinase B (AKT) signaling [49]. Specifically, miR-184 maintains SHIP2 levels through miR-205 expression [50]. This action assesses its effects on cytoskeletal remodeling, cell adhesion, and spreading, owing to the downregulation of miR-205. MiR-205 raises filamentous actin and p-cofilin via Rho activation; therefore, its downregulation results in central control of actin dynamics and cell motility [51]. It is also involved in keratinocytes’ survival function through two signaling pathways acting on p-Akt: inactivation of SHIP2 and activation of the phosphor-ezrin/radixin/moesin (p-ERM) [36,52]. MiR-122 is another RNA involved in cells migration owing to its influence on both the mitogen-activated protein kinase (MAPK) pathway and focal adhesion (CAM, cells adhesion molecules) [29,53]. Numerous 99- family miRNAs influence mTOR (mechanistic target of rapamycin kinase) by PI3K/AKT regulation, the upstream pathway [54]. PI3K/AKT pathway is also induced by mi-RNA26a [55]. Jiang et al. reported that miR-26a induces the expression of ITGA5 by upregulating the PI3K/AKT pathway [25]. Principally, through the integrin subunit alpha 5 (ITGA5) downregulation, miR-26a inhibits keratinocytes migration’s decreasing and therefore wound healing. This system permits the triggering of TGF-β1, thus cellular migration. Remarkably, the integrin subunit alpha 5 (ITGA5) overexpression could oppose the inhibitory effect of miR-26a [56]. miR-99 family members regulate cell proliferation, apoptosis, and migration [57]. Li et al. found that miR-222 influences the Wingless-related integration site (Wnt) signal, by targeting multiple genes, for example, Dickkopf-related protein 2 (DDK2), axin-like protein 2 (AXIN2), and FRAT regulator of WNT signaling pathway 2 (FRAT2) [29]. The MAPK pathway is induced by both miR-222 and miR-149 [58]. MiR-21 has been correlated with repression of growth, increased apoptosis, and cell cycle by its downregulation [59]. Moreover, miR-21 is involved with miR-203 in leptin receptor (LepR) regulation and, together with MiR-130a and miR-203, regulates early growth response 3 (EGR3) expression [32]. Other authors highlighted the action of miR-203 on inhibiting the proliferation process of epidermal stem cells in chronic ulcers [29,30,32,33,34,60]. Probably when miR-203 is expressed, tumor protein p63 (P63) and protein K15 (K15) and integrin B levels decreased remarkably [13]. Furthermore, overexpression of this miRNA decreases the expression of Rho-associated coiled-coil-containing protein kinase 2 (ROCK2), MAPK8, MAPK9, and protein kinase C alpha (PRKCA) by altering the Wnt/B-catenin signaling pathway [61]. Yang et al. demonstrate that miR-21 promotes keratinocyte migration and re-epithelialization of wounding inhibiting, respectively, the expression of metalloproteinase-3 (TIMP3) and T-cell lymphoma invasion and metastasis 1 (TIAM1) [35]. TGF-β-induced miR-21 expression leads to a significant tissue contraction, and in addition, it accelerates re-epithelialization [14]. Moreover, miR-21 has been linked to the activation of keloid fibroblasts and fibrosis [62]. MiR-203 has an important role in early inflammation and angiogenesis by controlling pro-migratory and pro-proliferative factors such as p63, LASP1 (LIM and SH3 protein 1), ras-related nuclear protein (RAN), RAPH1 (Ras association (RalGDS/AF-6) and pleckstrin homology domains 1) [33,63]. Jin et al. proved a correlation between PI3K/AKT, mTOR, insulin-like growth factor 1 receptor (IGF1R), and multiple miRNAs: miR-152, miR-365, miR-125a, and b, miR-181d, miR-100, and miR-30c [26]. These miRNAs are the fundamental regulator of the mTOR and MAPK signaling pathway in the skin [64].

**Figure 2:**
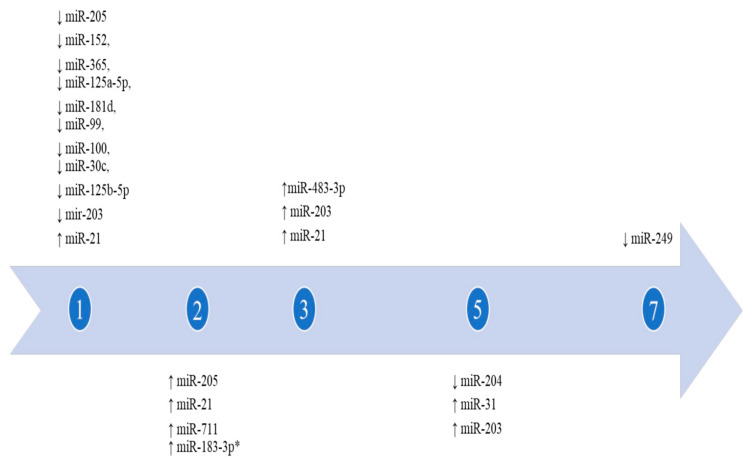
This figure represents the progressive expression of different miRNAs in time (days) after wounding. Only those miRNAs that are expressed according to a precise timeframe were considered. Only the first day of increase is shown. * miR-183-3p shows a different behavior between humans and rats; it was detected overexpressed in humans over 2 days and in rats over the course of 120 days. ↑ indicates up regulation, ↓ indicates down regulation.

In conclusion, by comparing the difference in the expression of miRNAs in the wound healing process, these biomarkers could become useful for the chronological diagnosis of the lesions [65], as shown in Figure 2.

Nevertheless, the reader should bear in mind that our work has some limitations. The pool of papers is heterogeneous, and it includes different kinds of studies, counting in vitro, in vivo, and human samples. Moreover, the small sample size (only 20 papers included) could interfere with the reliability of our findings. Certainly, more studies are needed in this field. Once the role of miRNAs in the wound healing process has been completely clarified, we hope to implement this knowledge in forensic applications soon thereafter. For future applications, it is conceivable to combine miRNAs analysis and histological and immunohistochemical tissue investigation, which have already been widely investigated in the forensic literature [1,2,3,4,6,7,8,9,10,11]. As in many other fields, miRNAs investigation carries great potential in forensic science [66,67,68]. A possible project could be an interlaboratory study to investigate the miRNAs differential expression in the same lesion and with the same analytical procedure, in addition to histological and immunohistochemical evaluation. The aim is to create a standardized protocol and start implementing these analyses since the reliability and reproducibility of analytical methodologies are the foundation for acceptance in legal proceedings.

## 5. Conclusions

Even if in the last years, some studies have provided new evidence about the forensic applications of miRNAs, much evidence still needs to be disclosed. MiRNAs are involved in many molecular pathways, and, owing to their ubiquity, they have been considered as markers in several fields. Herein, a compendium of the potential miRNAs that should be further investigated as wound vitality and age markers was provided. We found 20 articles on miRNAs involvement in skin wound and healing, but only 2 of them specifically referred to vitality demonstration in the forensic field. Further studies are certainly needed to deepen the role of miRNAs in inflammatory processes in wounded skin and to evaluate their reliability in distinguishing between antemortem and post-mortem lesions.

## Figures and Tables

**Table 1:** This table resumes all the studies implied in our revision. RT-PCR, when reported, is quantitative. Statistical analysis was performed in all studies except in Viticchiè et al., 2012, Yang et al., 2011, and Yu et al., 2010. AM, antemortem; H, histology; HaCaT, human keratinocyte cell line; HCECs, corneal epithelial cells; HADSCs, human subcutaneous adipose tissue; HEKs, human keratinocytes; HSF, human skin fibroblasts; IHC, immunohistochemistry; IHF, immunohistofluorescence; ISH, hybridization in situ; MP, morphometric analysis; NP, not performed; NS, not specified; PM, post-mortem; PMC, phase contrast morphometry; RT-PCR, reverse transcription–polymerase chain reaction; WB, Western blot. Excisional wounds refer to the removal of the skin surface, while incisional wounds represent a linear cut on a cell layer. ↑ indicates up regulation, ↓ indicates down regulation.

References	N. Cases	Model	Cells and/or Animals	Kind of Lesions	Controls	miRNAs	miRNAs Expression	Performed Analysis	RNA Isolation/cDNA Synthesis
Bertero et al., 2011 [21]	6	In vitro + in vivo	NHKs, HaCat + Mice	Scratch wound + Excisional wound	Normal skin	miR-483-3p	↑ 3d after injury infliction Peak: 6–7 days Normalization: at day 10 In epidermis (PM)	H + IHC + WB + RT-PCR	TRIzol reagent/NP
Cao et al., 2020 [22]	15	In vitro + in vivo	HaCaT, HSF + Mice	H_2_O_2_-induced wound + Excisional wound	Normal skin	miR-19b	↓ In epidermis if ↑ H_2_O_2_	H + IHC + WB + RT-PCR	TRIzol reagent/NP
Etich et al., 2017 [23]	15	In vivo	Mice	Excisional wound	Normal skin	miR-204	↓ from day 5 to day 10	H + RT-PCR	miRNeasy mini kit/miScript II RT kit
						miR-205	↓ from day 1 to day 7		
						miR-31	↑ In epidermis from day 5 to 14 after injury infliction (PM samples)		
He et al., 2020 [24]	NS	In vitro	HADSCs exosome, MALAT1 knockdown HaCaT and HSF	H_2_O_2_ induced scratch wound	Normal HaCaT and HSF	miR-124	↓ In epidermis if ↑ H_2_O_2_	WB + RT-PCR	TRIzol reagent/cDNA Synthesis SuperMix
Ibrahim et al., 2019 [18]	18	In vivo	Rats	Incisional wound	Normal skin	miR-205, miR-21	No statistical significance In epidermis 0, 24, and 48 h after death	H + RT-PCR	mirVana PARIS kit/NP
Jiang et al., 2020 [25]	3	In vitro	TGF-β1-treated HaCaT	Scratch wound	HaCaT	miR-26a	↓ In keratinocytes	WB + RT-PCR	EZ-Magna RIP kit/miScript II RT kit
Jin et al., 2013 [26]	5	In vivo + in vitro	HaCaT + Mice	Scratch wound + Excisional wound	Normal skin	miR-152, miR-365, miR-125a/b-5p, miR-181d, miR-99, miR-100, miR-30c	↓ In keratinocytes Peak: day 1 Normalization: day 5	WB + RT-PCR	TRIzol reagent/CyQUANT assay
Lang et al., 2017 [27]	9	In vitro + In vivo	HaCaT, HSF + Rats	Scratch wound + Excisional wound	No- TNFα-exposed cells	miR-149	↓ In epidermis	H + IHC + WB	mirVana miRNA isolation kit/NP
Lin et al., 2013 [28]	NS	In vitro	HCECs and HEKs	Scratch wound	Normal miR-16 levels	miR-205	↑ In corneal epithelial cells 24h after incision	WB + RT-PCR	TRIzol reagent/cDNA Synthesis SuperMix
Li et al., 2015 [29]	9	In vivo	Human (surgical samples)	Hypertrophic scar	Normal skin	miR-149, miR-203a, miR-222, miR-122	↓ In epidermis	ISH	miRcute RNA Isolation kit/NP
Liu et al., 2020 [30]	24	In vivo	Rats and DM rats	Excisional wound	No-diabetic rats	mir-203	↓ Over the course of the first 4 days in non-diabetic rats, over the course of the first 6 days in DM rats, in epidermis, dermis, and subcutaneous fat	H + IHC+ IHF + WB + RT-PCR	TRIzol rea-gent/RevertAid H minus first-strand cDNA synthesis kit
Lyu et al., 2018 [17]	22	In vivo	Mice	Burned skin	Normal skin	miR-135a-1-3p miR-183-3p miR-188-5p miR-3081-5p miR-5103 miR-6378 miR-6385 miR-6391 miR-6769b-5p miR-6969-5p miR-7005-5p miR-7036a-5p miR-7044-5p miR-710 miR-711 miR-7118-5p miR-7668-3p miR-8090 mmu-miR-874-3p	↑	H + RT-PCR	NS/PrimeScript RT reagent
						miR-155-5p miR-28a-3p miR-467b-3p miR-5132-5p miR-6924-3p	↓		
Long et al., 2018 [31]	NS	In vivo	miR-21 knock-in mice	Excisional wound	Normal skin	miR-21	↑	H + MP + ISH + RT-PCR	TaqMan microR-NA assay kit/NP
Neri et al., 2019 [19]	64	Autopsy casuistry	Human	Ligature marks	Normal skin	miR125a/b-5p miR130a-3p miR214-3p miR92a-3p	↑	H + IHC + RT-PCR	miScript miRNA PCR Array- Human Cell Differentiation/miScript II RT Kit
Pastar et al., 2012 [32]	21	In vivo + in vitro	HEK, HSF + Rats (6) + Humans (15)	Chronic ulcers + Excisional wound	Normal skin	miR-16, miR-20a, miR-21, miR-106a, miR-130a, miR-203	↑ In epidermis (miR-21: in epidermis, dermis, and blood vessels)	H + ISH + WB + RT-PCR	TaqMan Mi-croRNA As-says/NP
Viticchiè et al., 2012 [33]	NS	In vitro + in vivo	HEK and MEK + Mice	Excisional wound	Normal skin	miR-203	↑ In epidermis surrounding the wound and wound margin ↓ In keratinocytes at the migratory front (day 3 and 5 after wounding)	H + ISH + WB + RT-PCR	mirVana miRNA isolation kit/NP
Wang et al., 2012 [34]	NS	In vivo	Mice	Excisional wound	Normal skin	miR-31, miR-21, miR-203	↑ In epithelial cells	H + IHC + ISH + RT-PCR	TaqMan Mi-croRNA As-says/NP
						miR-249	↓ (day 7 after wounding)		
Yang et al., 2011 [35]	9	In vivo	Mice	Excisional wound	miR-21 knockdown mice	miR-21	↑ In epidermis after 0, 1, 2 and 3 days	H + IHC + IHF + RT-PCR	NS/NS
Yu et al., 2010 [36]	NS	In vitro	HCECs and HEKs (miR-205 and miR-184 downregulated)	Scratch wound	Normal cells	miR-205, miR-184	↑ In epithelial cells	IHC + PCM + WB	Quik-Change Site-Directed Muta-genesis kit/NP
Zhang et al., 2020 [37]	35	In vivo + autopsy cases	Mice (9) + autoptic human samples (26)	Excisional wound in burned skin	Mice unburned skin + mice PM burned skin	miR-711, miR-183-3p	↑ 48 h after excision in human burned skin (PM samples), 120 h in mice burned skin (AM samples)	RT-PCR	NS/PrimeScript RT reagent kit
Tot: 20 articles	Tot: 255 (at least)					Tot: 51 different miRNAs			

**Table 2:** This table shows the main characteristics of the studies included in our review. Each count refers only to the use of single method. Excisional wounds include one case of excisional wound in burned skin and 1 case of comparative study between chronic ulcers and excision. * Type of lesion refers only to in vivo studies. HE, hematoxylin and eosin staining; IHC, immunohistochemistry; IHF, immunohistofluorescence; ISH, hybridization in situ; MP, morphometric analysis; PMC, phase contrast morphometry; RT-PCR, real-time polymerase chain reaction; WB, Western blot.

Study’s Characteristics			N. of Studies (Tot. 20)	References
Model	In Vivo		8	[17,18,23,29,30,31,34,35]
	In Vitro		4	[24,25,28,36]
	In Vivo + In Vitro		6	[21,22,26,27,32,33]
	Human samples	Autoptic	2	[19,37]
		Surgical	1	[29]
Type of lesion *	Excisional wound		10	[21,22,23,26,27,30,31,33,34,35]
	Incisional wound		1	[18]
	Burned skin		1	[37]
	Ligature mark		1	[19]
	Chronic ulcer		1	[32]
	Hypertrophic scar		1	[29]
Performed analysis	RT-PCR		17	[17,18,19,21,22,23,24,25,26,28,30,31,32,33,34,35,37]
	Histology (HE)		13	[18,19,21,22,23,24,27,30,31,32,33,34,35]
	WB		11	[21,22,24,25,26,27,28,30,32,33,36]
	IHC		8	[19,21,22,27,30,34,35,36]
	ISH		5	[29,31,32,33,34]
	IHF		2	[30,35]
	MP		1	[31]
	PMC		1	[36]

**Table 3:** The list of miRNAs pinpointed by the included papers and the respective target genes, when available.

miRNAs	Target Genes and/or Proteins	References
miR19-b	CCL1, TGFβ	[22]
miR-21	Factor 3, vinculin, LepR, EGR3, Collagen, TGF-β, TIMP3, TIAM1, TP53	[29,32,34,35]
miR-26a	ITGA5, PI3K/ AKT, SMAD1, GSK3β	[25]
miR-30c	PI3K/AKT, mTOR, IGF1R	[26]
miR-31	IL-1b, PTPRC/CD45, SHIP2, RNU6B, Col1a1	[23]
miR-99	PI3K/AKT, mTOR, IGF1R	[26]
miR-100	PI3K/AKT, mTOR, IGF1R	[26]
miR-122	MAPK, lysosome, insulin signaling pathway, focal adhesion	[29]
miR-125a-5p, miR-125b-5p	PI3K/AKT, mTOR, IGF1R	[26]
miR-130a	EGR3	[32]
miR-149	IL-1a, IL-1b, IL-6, TGF-β3, collagen III, Nf-Kb, RelB, Rel, MAPK	[27,29]
miR-152	PI3K/AKT, mTOR, IGF1R	[26]
miR-181d	PI3K/AKT, mTOR, IGF1R	[26]
miR-184	SHIP2, PI3K-Akt, actin filaments, p-cofilin (via Rho)	[36]
miR-203	MAPK, lysosome, insulin signaling pathway, focal adhesion, K15, P63, integrin-β1, TCF-4, ID-2, CD44, VEGFA, NRCAM, C-MET, Wnt, Notch, Factor 3, vinculin, LepR, EGR3, p63, LASP1, RAN, RAPH1	[29,30,32,33]
miR-204	IL-1b, PTPRC/CD45, SHIP2, RNU6B, Col1a1	[23]
miR-205	IL-1b, PTPRC/CD45, SHIP2, RNU6B, Col1a1, KCNJ10, SHIP2, PI3K-Akt, actin filaments, p-cofilin (via Rho), p-ERM	[23,28,36]
miR-222	DDK2, AXIN2, FRAT2, MAPK	[29]
miR-365	PI3K/AKT, mTOR, IGF1R	[26]
miR-483-3p	MK2, YAP1, ASH2, MKI67	[21]

## Data Availability

Not applicable.
